# Supplementary material for: Physical Movement Habit Formation in Sedentary Office Workers: Protocol Paper
Source: Methods Protoc. 2022 Nov 25;5(6):94. doi: 10.3390/mps5060094 (PMC9781315; doi:10.3390/mps5060094)
Supplement: Supplementary file 1 [file mps-05-00094-s001.zip › mps-1950974-supplementary.pdf]

The Formation of Physical Movement Habits within the Workplace

Description

Within industrialised societies, health-risk behaviours, such as high levels of sedentary behaviour, have been identified as leading causes of disease-related preventable deaths (Amler & Eddins, 1987; Conner & Norman, 2015; Mokdad et al., 2016; Noble, Paul, Turon, & Oldmeadow, 2015; Nylander, 2016; Organization, 2015). With the increase in technology and decrease in physically demanding jobs, high levels of sedentary behaviour and a general disengagement in physical activity are major global health concerns (Borodulin, Laatikainen, Juolevi, & Jousilahti, 2008; Brownson, Boehmer, & Luke, 2005; Bull et al., 2020; Kirk & Rhodes, 2011; Matthews et al., 2008; Organization, 1998; Straker & Mathiassen, 2009). A particular subset of the population at risk of these concerns are those working in highly sedentary jobs, such as office workers. Office workers are at particularly high risk for developing conditions related to sedentary behaviour (Emanuele, 2008; Hemingway, Shipley, Stansfeld, & Marmot, 1997; Higham, 2019; Hitosugi, Niwa, & Takatsu, 2000; Loh & Redd, 2008). Also, as office workers often spend a large proportion of their day engaging in sedentary behaviour at work, the workplace has become an opportunistic environment for targeting health-related behaviours (Dugdill, Brettle, Hulme, McCluskey, & Long, 2008; Parry & Straker, 2013; Rhodes & De Bruijn, 2010; Salmon, Owen, Crawford, Bauman, & Sallis, 2003).

Social cognition theories, such as the Theory of Planned Behaviour (TPB –Ajzen, 1991), have been applied widely to understand the determinants of sedentary behaviour and physical activity (McEachan, Conner, Taylor, & Lawton, 2011; Prapavessis, Gaston, & DeJesus, 2015). Central to many of these models is the premise that intention, which is assumed to be guided by conscious deliberation, is the most proximal predictor of behaviour, with the stronger the intention the more likely the behaviour will be acted on (Ajzen, 1991; Armitage & Conner, 2001; Hagger, Chatzisarantis, & Biddle, 2002; Hagger, Cheung, Ajzen, & Hamilton, 2022; Hamilton, van Dongen, & Hagger, 2020; McEachan et al., 2011; Symons Downs & Hausenblas, 2005). However, it is well documented that the link between intention and behaviour is not perfect and that there remains an intention-behaviour gap (Rhodes & de Bruijn, 2013). More recently, literature has looked at incorporating constructs that underpin nonconscious processes, such as habit and implicit attitudes, into models predicting sedentary behaviour and physical activity and found these more automatic, nonconscious constructs to be important contributors to behaviour (Brand & Ekkekakis, 2018; Conroy, Maher, Elavsky, Hyde, & Doerksen, 2013; Gardner, de Bruijn, & Lally, 2011; Phipps, Hannan, Rhodes, & Hamilton, 2021; Rhodes, 2021; Rollo, Gaston, & Prapavessis, 2016).

Habit is defined as a psychological construct that is dependent on associations forming between consistent contextual cues and repeated behaviours (Hagger, 2019; Lally & Gardner, 2013; Webb & de Bruin, 2020). A key feature of habitual actions is that they require less cognitive processing in comparison to intentional behaviours, making the behaviour more accessible to perform (Förster & Jostmann, 2015; Wood, 2017). By focusing on forming habitual responses to cues within the environment that promote physical

movement (i.e., stairs), one can increase the amount of movement performed during the day, thus limiting sedentary behaviour without taxing cognitive load (Hamilton, Fraser, & Hannan, 2019). Furthermore, as simple behaviours are more conducive to habit formation, they also require less time to reach habit plateau (Lally et al., 2010). As time constraints are often an influencing factor for attrition in physical activity interventions, this structure offers a unique method to counter this barrier (Dugdill et al., 2008; Malik, Blake, & Suggs, 2014; Salmon et al., 2003).

Literature looking at developing interventions that are aimed at developing habits around performing physical movement should focus on four key aspects: the target behaviour is personally relevant; the target behaviour is considered a simple change to implement within pre-existing practices; the target behaviour is realistic to action; and the target behaviour can be monitored and assessed against behavioural goals (Gardner, Lally, & Rebar, 2020; Gardner et al., 2014). Furthermore, when developing interventions, previous literature has looked at implementation techniques, comparing internet-based to in person delivery modes. While there appears to be advantages and disadvantages for each method, an internet-based delivery mode offers potential methods for countering common barriers associated with physical activity interventions, such as time constraints. As these barriers contribute to poor attrition rates within physical activity interventions, online interventions provide flexible delivery modes with the added opportunity to reach wider populations (Cugelman, Thelwall, & Dawes, 2011; Wantland, Portillo, Holzemer, Slaughter, & McGhee, 2004).

Study Information

Hypotheses

The current study aims to develop an online habit-based intervention designed to reduce sedentary behaviour within the workplace, using environmental cues to instigate simple behavioural changes. Four hypotheses are preregistered for the current study. Based on previous research, the primary objective is to test the effectiveness of the intervention on limiting occupational sedentary behaviour and increasing occupational physical movement habits within the workplace. It is hypothesised that reported occupational sedentary behaviour will have a decrease (H1) and reported occupational physical movement will have an increase (H2) across baseline (T1) and follow up points two weeks post baseline (T2) and four weeks post baseline (T3) in comparison to those in the control group. It is further expected that occupational physical movement habit will have an increase (H3) and occupational sedentary behaviour will have a decrease (H4) across baseline (T1) and follow up points two weeks post baseline (T2) and four weeks post baseline (T3).

Design Plan

Study type

Experiment - A researcher randomly assigns treatments to study subjects, this includes field or lab experiments. This is also known as an intervention experiment and includes randomized controlled trials.

Blinding

- For studies that involve human subjects, they will not know the treatment group to which they have been assigned.

Is there any additional blinding in this study?

No response

Study design

The study is a 2 (condition / group) x 3 (time) mixed-model randomised control trial, where participants are randomly assigned to an active control (education) arm or an experimental (education and habit). The intervention is designed to reduce occupational sedentary behaviour by forming habits towards occupational physical movement within the workplace environment through the use of an online intervention. Condition is the between participants variable and time (baseline, 2-week follow up, and 4-week follow up) is the within participants variable.

Control Group. Participants in the control group are provided with an information sheet adapted from the World Health Organisation recommendations on physical activity as well as the Canadian guidelines on sedentary behaviour (Ross et al., 2020). The World Health Organisation recommends adults should aim to do more than 150 minutes of moderate to vigorous physical activity over the week (Bull et al., 2020). Canadian guidelines for sedentary behaviour involve limiting sedentary time to eight hours or less and breaking up long periods of sitting as often as possible, along with replacing sedentary behaviour with additional physical activity (Ross et al., 2020). The information pertains to the risks of increased sedentary behaviour and the health benefits of increased movement during the day.

Intervention Group. Along with the information sheet, participants allocated to the intervention group will be provided with a digital poster outlining the positive normative beliefs of other office workers towards movement in the workplace. This poster is developed based on previous literature (Hamilton et al., 2019) and guided by literature on the social norms approach (Dempsey, McAlaney, & Bewick, 2018; McAlaney, Bewick, & Hughes, 2011; Perkins, 1997, 2003; Prapavessis et al., 2015). Along with this poster, participants are provided with the '10 Top Tips' poster, which includes 10 simple behaviours that can be performed within the workplace to increase movement during the day. Participants are prompted to choose tips that they are confident they could perform and write an action plan outlining where, when and how they intend to implement their chosen tips, along with a coping plan of how they intend to overcome potential barriers to behavioural production. Following this, participants will be asked to write an encouraging statement to instigate their self-efficacy towards achieving their tips. Furthermore, participants will be provided with a self-monitoring tick sheet that can be used to monitor their behavioural progress. The intervention group is encouraged to print their action and coping plans as reminders of their intentions and to email the self-monitoring tick sheet to the first researcher at the end of each week.

Randomization

We will use simple randomization, where each participant will be randomly allocated to one of the two groups. The randomization will be conducted by the Qualtrics randomizer feature following completion of the pre-intervention survey. The Qualtrics randomizer operates using a Mersenne Twister pseudorandom number generator which is seeded using a Unix timestamp (in milliseconds). By nature of this method of random assignment, the sequence will not be determined until the participant is assigned.

Sampling Plan

Existing Data

Registration prior to creation of data

Explanation of existing data

No response

Data collection procedures

Participants are recruited via the Griffith University email broadcast, University networks and through social media advertisement. Participants are included if they are aged 18 years or older; self-describe as having a highly sedentary job (i.e., sit for at least 75% of the working day); and work full-time from either a commercial office, home office, or a combination of the two. Participants are excluded if they self-describe as not wanting to limit their sedentary behaviour at work.

Sample Size

Our target sample size at the 4-week follow-up is 180. To allow for 25% attrition, we will attempt to recruit 226 participants at baseline (T1).

Sample size rationale

To test intervention effects, there is an intended recruitment of 226 participants. Based on previous research in this field and ongoing research within this population group (Andersen et al., 2013), it is anticipated that there will be approximately a 25% attrition over the four weeks of follow-up for reasons such as changes in job status, vacancy and failure to complete questionnaires. A total sample of approximately 180 completing participants (90/group) is required to detect a medium effect in habit development towards occupational physical movement. This sample was calculated by a power analysis using WebPower program (Zhang & Yuan, 2018). Significance level (alpha) was established at 0.05 to avoid a Type 1 error and effect size was determined at $f = .35$. This means that for a 95% chance of detecting a significant intervention effect at a four-week follow-up, approximately 90 participants are needed in each group.

Variables

Manipulated variables

Habit in relation to the target behaviour of limiting occupational sedentary behaviour and increasing occupational physical movement as part of the daily work routine will be manipulated in the intervention. Participants will be asked to complete measures of social demographic factors (Time 1 only) and social psychological and behavioural measures in relation to the target behaviour at Time 1, Time 2 and Time 3 to evaluate change over the 4-week period.

Measured variables

The primary outcome variables will assess the effectiveness of the intervention on limiting occupational sedentary behaviour and developing habits towards occupational physical movement within the workplace. The Occupational Sitting and Physical Activity Questionnaire (OSPAQ) will be used to measure occupational sedentary behaviour and occupational physical movement. The OSPAQ has been validated for use with both commercial office and home office working populations (Chau, Van Der Ploeg, Dunn, Kurko, & Bauman, 2012; Dillon et al., 2021; Jancey, Tye, McGann, Blackford, & Lee, 2014). To measure the construct of habit for occupational sedentary behaviour and physical movement the four-item Self-Report Behavioural Automaticity Index was used (Gardner, Abraham, Lally, & de Bruijn, 2012). For occupational physical movement habit, participants will be asked to rate their agreement with the following statements "Do you agree that doing occupational physical movement as part of your daily work routine is something: I do automatically; I do without having to consciously remember; I do without thinking; I start doing before I realise I am doing it", measured on a 7-point Likert scale (1 = strongly disagree, 7 = strongly agree). To measure habit to engage in occupational sedentary behaviour, participants will be asked to rate their agreeance with the following statements " Do you agree that engaging occupational sedentary behaviour as part of your daily work routine is something: I do automatically; I do without having to consciously remember; I do without thinking; I start doing before I realise I am doing it", measured on a 7-point Likert scale (1 = strongly disagree, 7 = strongly agree). Similar items have been used in previous research (Hamilton et al., 2019; Phipps, Hagger, & Hamilton, 2020)

Indices

Mean average of the items scores for the behaviour and habit variables will be computed. Higher mean scores will indicate greater participation in the target behaviour and greater endorsement of the habit construct with reference to the target behaviour.

Analysis Plan

Statistical models

To assess the effect of the intervention on reducing occupational sedentary behaviour and increasing occupational physical movement; as well as the interventions effect on increasing occupational physical movement habit and its effects on decreasing occupational sedentary behaviour, four mixed methods 2 (condition / group) x 3 (time) ANOVA's will be conducted. To assess the overall effects of the intervention, group (control or intervention) will be used as the between-subjects independent variable; time (baseline/T1, T2 and T3) will be used as

the within-subjects independent variable with occupational sedentary behaviour, occupational physical movement, occupational physical movement habit and occupational sedentary behaviour habit as the dependent variables. Each ANOVA will use an adjusted alpha level of .01 to protect from inflation of type I error. Results, however, will report on any effects using the conventionally accepted significance cut-off level of .05 as well as the pre-specified cut-off level of .01. Where an ANOVA indicates a significant time*group interaction for either of the dependent variables, a simple effects analysis will be conducted.

Transformations

Where variables meet criteria for significant skewness (ratio of skew to SE greater than 3.29) or significant kurtosis (ratio of kurtosis to SE greater than 3.29), a square root transformation will be conducted on the variable in question. If this transformation does not correct the skewness or kurtosis, a logarithmic transformation will be applied to the variable. If this again does not resolve the skewness or kurtosis, an inverse transformation will be applied. Analyses will be conducted using the final transformation of the variable in question and compared to the analyses using the variable prior to transformation. The analysis using the transformed variable will only be retained and reported as the primary analysis if it changes the conclusion regarding significance of the results. All analyses will be reported.

Inference criteria

Hypotheses will be evaluated using null hypothesis significance tests (p-values). Because we are conducting several tests, we will adjust the critical value of α to .01 for determining if ANOVA and follow-up tests suggest that the results are significantly different from those expected if the null hypothesis were correct.

Data exclusion

Participants will be asked a question to detect careless responding (e.g. please select option 3 to ensure you are paying attention). Participants who do not answer this question correctly will be excluded from analysis.

Missing data

Missing data will be imputed using the Expectation-Maximisation (E-M) algorithm.

Exploratory analysis

No response

Other

Ajzen, I. (1991). The theory of planned behavior. Organizational behavior and human decision processes, 50(2), 179-211.
Amler, R. W., & Eddins, D. L. (1987). Cross-sectional analysis: precursors of premature death in the United States. Am J Prev Med, 3(Suppl. 5), 181-187.
Andersen, L. L., Sundstrup, E., Boysen, M., Jakobsen, M. D., Mortensen, O. S., & Persson, R. (2013). Cardiovascular health effects of internet-based encouragements to do daily

workplace stair-walks: randomized controlled trial. Journal Of Medical Internet Research, 15(6), e127.
Armitage, C. J., & Conner, M. (2001). Efficacy of the theory of planned behaviour: A meta-analytic review. British journal of social psychology, 40(4), 471-499.
Borodulin, K., Laatikainen, T., Juolevi, A., & Jousilahti, P. (2008). Thirty-year trends of physical activity in relation to age, calendar time and birth cohort in Finnish adults. European Journal Of Public Health, 18(3), 339-344.
Brand, R., & Ekkekakis, P. (2018). Affective–reflective theory of physical inactivity and exercise. German Journal of Exercise and Sport Research, 48(1), 48-58.
Brownson, R. C., Boehmer, T. K., & Luke, D. A. (2005). Declining rates of physical activity in the United States: what are the contributors? Annu. Rev. Public Health, 26, 421-443.
Bull, F. C., Al-Ansari, S. S., Biddle, S., Borodulin, K., Buman, M. P., Cardon, G., . . . Chou, R. (2020). World Health Organization 2020 guidelines on physical activity and sedentary behaviour. British Journal of Sports Medicine, 54(24), 1451-1462.
Chau, J. Y., Van Der Ploeg, H. P., Dunn, S., Kurko, J., & Bauman, A. E. (2012). Validity of the occupational sitting and physical activity questionnaire. Medicine and science in sports and exercise, 44(1), 118-125.
Conner, M., & Norman, P. (2015). EBOOK: Predicting and Changing Health Behaviour: Research and Practice with Social Cognition Models: McGraw-Hill Education (UK).
Conroy, D. E., Maher, J. P., Elavsky, S., Hyde, A. L., & Doerksen, S. E. (2013). Sedentary behavior as a daily process regulated by habits and intentions. Health Psychology, 32(11), 1149.
Cugelman, B., Thelwall, M., & Dawes, P. (2011). Online interventions for social marketing health behavior change campaigns: a meta-analysis of psychological architectures and adherence factors. Journal Of Medical Internet Research, 13(1), e17.
Dempsey, R. C., McAlaney, J., & Bewick, B. M. (2018). A critical appraisal of the social norms approach as an interventional strategy for health-related behavior and attitude change. Frontiers in Psychology, 9, 2180.
Dillon, K., Hiemstra, M., Mitchell, M., Bartmann, N., Rollo, S., Gardiner, P. A., & Prapavessis, H. (2021). Validity of the occupational sitting and physical activity questionnaire (OSPAQ) for home-based office workers during the COVID-19 global pandemic: A secondary analysis. Applied Ergonomics, 97, 103551.
Dugdill, L., Brettle, A., Hulme, C., McCluskey, S., & Long, A. (2008). Workplace physical activity interventions: a systematic review. International Journal of Workplace Health Management.
Emanuele, P. (2008). Deep vein thrombosis. Aaohn Journal, 56(9), 389-394.
Förster, J., & Jostmann, N. B. (2015). What is automatic self-regulation? Zeitschrift für Psychologie.
Gardner, B., Abraham, C., Lally, P., & de Bruijn, G.-J. (2012). Towards parsimony in habit measurement: Testing the convergent and predictive validity of an automaticity subscale of the Self-Report Habit Index. International Journal of Behavioral Nutrition and Physical Activity, 9(1), 1-12.

Gardner, B., de Bruijn, G.-J., & Lally, P. (2011). A systematic review and meta-analysis of applications of the self-report habit index to nutrition and physical activity behaviours. Annals of Behavioral Medicine, 42(2), 174-187.
Gardner, B., Lally, P., & Rebar, A. L. (2020). Does habit weaken the relationship between intention and behaviour? Revisiting the habit-intention interaction hypothesis. Social and Personality Psychology Compass, 14(8), e12553.
Gardner, B., Thuné-Boyle, I., Iliffe, S., Fox, K. R., Jefferis, B. J., Hamer, M., . . . Wardle, J. (2014). 'On Your Feet to Earn Your Seat', a habit-based intervention to reduce sedentary behaviour in older adults: study protocol for a randomized controlled trial. Trials, 15(1), 1-13.
Hagger, M., Chatzisarantis, N., & Biddle, S. (2002). A meta-analytic review of the theories of reasoned action and planned behavior in physical activity: Predictive validity and the contribution of additional variables. Journal Of Sport & Exercise Psychology.
Hagger, M. S. (2019). Habit and physical activity: Theoretical advances, practical implications, and agenda for future research. Psychology of sport and exercise, 42, 118-129.
Hagger, M. S., Cheung, M. W.-L., Ajzen, I., & Hamilton, K. (2022). Perceived behavioral control moderating effects in the theory of planned behavior: A meta-analysis. Health Psychology.
Hamilton, K., Fraser, E., & Hannan, T. (2019). Habit-based workplace physical activity intervention: a pilot study. Occupational medicine, 69(7), 471-474.
Hamilton, K., van Dongen, A., & Hagger, M. S. (2020). An extended theory of planned behavior for parent-for-child health behaviors: A meta-analysis. Health Psychology, 39(10), 863.
Hemingway, H., Shipley, M. J., Stansfeld, S., & Marmot, M. (1997). Sickness absence from back pain, psychosocial work characteristics and employment grade among office workers. Scandinavian Journal Of Work, Environment & Health, 121-129.
Higham, W. (2019). The Work Colleague of the Future A report on the long-term health of office workers. no. June.
Hitosugi, M., Niwa, M., & Takatsu, A. (2000). Rheologic changes in venous blood during prolonged sitting. Thrombosis research, 100(5), 409-412.
Jancey, J., Tye, M., McGann, S., Blackford, K., & Lee, A. H. (2014). Application of the Occupational Sitting and Physical Activity Questionnaire (OSPAQ) to office based workers. Bmc Public Health, 14. doi:10.1186/1471-2458-14-762
Kirk, M. A., & Rhodes, R. E. (2011). Occupation correlates of adults' participation in leisure-time physical activity: a systematic review. American Journal Of Preventive Medicine, 40(4), 476-485.
Lally, P., & Gardner, B. (2013). Promoting habit formation. Health Psychology Review, 7(sup1), S137-S158.
Lally, P., Van Jaarsveld, C. H., Potts, H. W., & Wardle, J. (2010). How are habits formed: Modelling habit formation in the real world. European journal of social psychology, 40(6), 998-1009.
Loh, K., & Redd, S. (2008). Understanding and preventing computer vision syndrome. Malaysian family physician: the official journal of the Academy of Family Physicians of Malaysia, 3(3), 128.

Malik, S. H., Blake, H., & Suggs, L. S. (2014). A systematic review of workplace health promotion interventions for increasing physical activity. British Journal of Health Psychology, 19(1), 149-180.
Matthews, C. E., Chen, K. Y., Freedson, P. S., Buchowski, M. S., Beech, B. M., Pate, R. R., & Troiano, R. P. (2008). Amount of time spent in sedentary behaviors in the United States, 2003–2004. American Journal Of Epidemiology, 167(7), 875-881.
McAlaney, J., Bewick, B., & Hughes, C. (2011). The international development of the 'Social Norms' approach to drug education and prevention. Drugs: education, prevention and policy, 18(2), 81-89.
McEachan, R. R. C., Conner, M., Taylor, N. J., & Lawton, R. J. (2011). Prospective prediction of health-related behaviours with the theory of planned behaviour: A meta-analysis. Health Psychology Review, 5(2), 97-144.
Mokdad, A. H., Forouzanfar, M. H., Daoud, F., Mokdad, A. A., El Bcheraoui, C., Moradi-Lakeh, M., . . . Cercy, K. (2016). Global burden of diseases, injuries, and risk factors for young people's health during 1990–2013: a systematic analysis for the Global Burden of Disease Study 2013. The Lancet, 387(10036), 2383-2401.
Montori, V. M., & Guyatt, G. H. (2001). Intention-to-treat principle. Cmaj, 165(10), 1339-1341.
Noble, N., Paul, C., Turon, H., & Oldmeadow, C. (2015). Which modifiable health risk behaviours are related? A systematic review of the clustering of Smoking, Nutrition, Alcohol and Physical activity ('SNAP') health risk factors. Preventive Medicine, 81, 16-41.
Nylander, C. (2016). Protective factors, health-risk behaviours and the impact of coexisting ADHD among adolescents with diabetes and other chronic conditions. Acta Universitatis Upsaliensis,
Organization, W. H. (1998). Obesity: preventing and managing the global epidemic: report of a WHO consultation on obesity, Geneva, 3-5 June 1997. Retrieved from
Organization, W. H. (2015). World health statistics 2015: World Health Organization.
Parry, S., & Straker, L. (2013). The contribution of office work to sedentary behaviour associated risk. Bmc Public Health, 13(1), 1-10.
Perkins, H. W. (1997). College student misperceptions of alcohol and other drug norms among peers: Exploring causes, consequences, and implications for prevention programs. Designing alcohol and other drug prevention programs in higher education: Bringing theory into practice, 177-206.
Perkins, H. W. (2003). The emergence and evolution of the social norms approach to substance abuse prevention. The social norms approach to preventing school and college age substance abuse: A handbook for educators, counselors, and clinicians, 3-17.
Phipps, D. J., Hagger, M. S., & Hamilton, K. (2020). Predicting limiting 'free sugar'consumption using an integrated model of health behavior. Appetite, 150, 104668.
Phipps, D. J., Hannan, T. E., Rhodes, R. E., & Hamilton, K. (2021). A dual-process model of affective and instrumental attitudes in predicting physical activity. Psychology of sport and exercise, 54, 101899.
Prapavessis, H., Gaston, A., & DeJesus, S. (2015). The Theory of Planned Behavior as a model for understanding sedentary behavior. Psychology of sport and exercise, 19, 23-32.

Rhodes, R. E. (2021). Multi-Process Action Control in Physical Activity: A Primer. Frontiers in Psychology, 12, 797484-797484.
Rhodes, R. E., & De Bruijn, G.-J. (2010). Automatic and motivational correlates of physical activity: Does intensity moderate the relationship? Behavioral Medicine, 36(2), 44-52.
Rhodes, R. E., & de Bruijn, G. J. (2013). How big is the physical activity intention–behaviour gap? A meta-analysis using the action control framework. British Journal of Health Psychology, 18(2), 296-309.
Rollo, S., Gaston, A., & Prapavessis, H. (2016). Cognitive and motivational factors associated with sedentary behavior: a systematic review. Aims Public Health, 3(4), 956.
Ross, R., Chaput, J.-P., Giangregorio, L. M., Janssen, I., Saunders, T. J., Kho, M. E., . . . McLaughlin, E. C. (2020). Canadian 24-Hour Movement Guidelines for Adults aged 18–64 years and Adults aged 65 years or older: an integration of physical activity, sedentary behaviour, and sleep. Applied physiology, nutrition, and metabolism, 45(10), S57-S102.
Salmon, J., Owen, N., Crawford, D., Bauman, A., & Sallis, J. F. (2003). Physical activity and sedentary behavior: a population-based study of barriers, enjoyment, and preference. Health Psychology, 22(2), 178.
Schulz, K. F., Altman, D. G., & Moher, D. (2010). CONSORT 2010 statement: updated guidelines for reporting parallel group randomised trials. Trials, 11(1), 1-8.
Straker, L., & Mathiassen, S. E. (2009). Increased physical work loads in modern work–a necessity for better health and performance? Ergonomics, 52(10), 1215-1225.
Symons Downs, D., & Hausenblas, H. (2005). Exercise behavior and the theories of reasoned action and planned behavior: A meta-analytic update. Journal of physical activity and health, 2, 76-97.
Wantland, D. J., Portillo, C. J., Holzemer, W. L., Slaughter, R., & McGhee, E. M. (2004). The effectiveness of Web-based vs. non-Web-based interventions: a meta-analysis of behavioral change outcomes. Journal Of Medical Internet Research, 6(4), e40.
Webb, T., & de Bruin, M. (2020). Monitoring interventions. Handbook of behavior change, 537-553.
Wood, W. (2017). Habit in personality and social psychology. Personality and social psychology review, 21(4), 389-403.
Zhang, Z., & Yuan, K.-H. (2018). Practical statistical power analysis using Webpower and R: Isdsa Press.
